# Socio-economic differences among low-birthweight infants in Hungary. Results of the Cohort ‘18 –Growing Up in Hungary birth cohort study

**DOI:** 10.1371/journal.pone.0291117

**Published:** 2023-09-01

**Authors:** Laura Szabó, Julianna Boros

**Affiliations:** 1 Hungarian Demographic Research Institute, Budapest, Hungary; 2 Institute of Behavioural Sciences, Semmelweis University, Budapest, Hungary; Harvard T.H. Chan School of Public Health, UNITED STATES

## Abstract

**Background:**

As Hungary had the fifth-highest rate of low-birthweight (LBW) in the EU27 in 2020, LBW still remains a public health problem for the country.

**Objective:**

Our goal is to examine whether LBW in Hungary is determined by the mothers’ educational attainment, adjusted for other maternal characteristics (SES, health behaviour and psychological status during pregnancy) among mothers who gave birth in 2018–2019 in Hungary.

**Methods:**

Source of data is the first and second wave of the Cohort ‘18 –Growing Up in Hungary longitudinal birth cohort study (n = 8185). It is based on a nationwide representative sample of pregnant women who gave birth between March 2018 and April 2019. All data were self-reported by mothers. We examined the association between maternal educational attainment and the risk of giving birth to an LBW-child (<2500g) by using logistic regression analysis. The highest educational attainment of the mother is measured by a five-value categorical variable (ISCED 97: 0–1; 2; 3C; 3–4; 5–6).

**Results:**

5.9% of women had LBW children. This rate is 18.0% among the lowest educated women with ISCED 97: 0–1; and it is 3.6% among the highest educated women with ISCED 97: 5–6. The adjusted predicted probabilities of LBW for these two groups of women are 13.5%, and 3.4% respectively, adjusted for household income quantiles, Roma ethnic background, residence place, smoking, alcohol consumption, and depression during pregnancy; controlled for mother’s height, age at birth, parity and child’s sex. Compared to women with the lowest level of education, the risk of giving birth to an LBW child decreases by 34.6% for those with the second level of education, by 60.1% for those with the third level of education, by 72.5% for those with the fourth level of education and by 77.2% for those with the highest level of education. Smoking during pregnancy significantly increases the risk of giving birth to an LBW by 54.9%. Being depressed at 7^th^ month of pregnancy decreased the risk of giving birth to an LBW child in our sample by 13.2%, however the relationship is not significant.

**Conclusion:**

Our analysis confirmed that maternal educational attainment has a significant impact on the risk of LBW net of by other maternal SES and health behaviour factors. Nevertheless, even after adjusting for these covariates, inequality in LBW by maternal educational attainment persists.

## Introduction

The proportion of low-birthweight (<2500g) infants in Hungary is high in international comparison: in 2018 it was 8.5% (it was still 7.7% in 2020), and the time series show no considerable decrease (S1 Fig). If we examine the vital statistics of the Hungarian Central Statistical Office (HCSO) regarding the proportion of LBW infants in Hungary from the perspective of maternal educational attainment, we find that those with at least vocational education are relatively clearly separated from those with at most primary education, and this difference has not declined significantly over the past three decades. In 2018, only 6% of mothers with tertiary education gave birth to LBW infants in Hungary, compared to 18% of those who had completed less than eight years of primary school in 2018 (S2 Fig). But what is the relationship between maternal educational attainment and LBW? Our aim is to examine how mothers’ educational attainment influences children’s birth weight, focusing on mothers who gave birth to a child in 2018–2019 in Hungary. Moreover, we are interested in looking at how this relationship is explained by other maternal socioeconomic (household income, ethnicity, residence place), health behaviour (smoking, alcohol consumption during pregnancy) and psychological factors (depression at 7^th^ month of pregnancy).

## Theoretical background

The negative health consequences of LBW are widely known, for example it greatly increases the chances of infant mortality [1–4]. Every year, 1.1 million babies worldwide die from complications of preterm birth (born before 37 weeks of gestation), the majority of them being also LBW child [1]. In addition to a higher risk of infant mortality, LBW can also increase the incidence of health problems in childhood and adulthood, such as disability, hospitalization and poorer mental development, as well as cardiovascular diseases or diabetes [5–8]. For this reason, one of the six targets of the *Comprehensive implementation plan on maternal*, *infant and young child nutrition* of the World Health Assembly (WHA) is to reduce the prevalence of LBW children by 30% between 2012 and 2025 [1].

At the global level, the vast majority of LBW births occur in low- and middle-income countries. However, even high-income countries with lower social inequality and universal access to health care face problems of LBW, especially among their most vulnerable populations [9]. Many biological, maternal lifestyle and socio-economic factors increase the risk of LBW [10] including medical risk before pregnancy (e.g. chronic hypertension, glucose metabolism disorders, autoimmune diseases or renal diseases [11]); problems of current pregnancy (e.g. gestational hypertension, gestational diabetes, weight gain, eating disorders, maternal malnutrition or short birth intervals [12]); environmental and behaviour risks (e.g. smoking, drug consumption, or exposure to toxic substances [13, 14]), inadequate prenatal care or socioeconomic risk factors [15]. The latter includes maternal educational attainment [11–12, 16–20], maternal age [12, 21–23], ethnicity [11, 24–26] and marital status [20]. Social inequalities in health thus appear in the very earliest stages of life [27–29].

Our analysis fits into this research framework. We use the social determinants of health model of Marmot as a starting point [30]: health status is influenced by social position (which includes maternal educational attainment, occupation, income and ethnicity), but this relationship is affected by the socioeconomic context on the one hand and material circumstances, behavioural and psychosocial factors on the other hand.

Focusing on socioeconomic risk factors, the role of education seems to be one of the most important determinant of LBW. Based on Silvestrin et al’s meta-analysis [16] of cohort and cross-sectional studies, high maternal education showed a 33% protective effect against LBW, whereas medium degree of education showed no significant protection when compared to low maternal education. Liu et al [18] investigated the causal association between educational attainment (EA) of parents and offspring birth weight (BW), and they found that BW is positively causally affected by maternal EA. Each one standard deviation (SD) increase in female EA was associated with 0.24 SD higher of offspring BW (95% confidence interval [CI], 0.10 to 0.37, p < 0.001). A meta-analysis of Shokri et al [12] showed that in Iran middle school and lower education risk for LBW vs. high school and higher was 1.56 (95% CI: 1.28–1.90, p < 0.001), while Nazari et al [31] found no significant differences in LBW by maternal educational levels of Iranian women. According to van den Berg et al’s analysis [19], lower educated women in the Netherlands had almost a two-fold risk for having a LBW child than higher educated women (OR = 1.98, 95% CI 1.35–2.89).

Panico et al’s analysis [26] compared the data of the French Longitudinal Study of Children (ELFE) in France and the Millennium Cohort Study (MCS) in the UK. The results showed that the differences in LBW by educational level followed slightly different patterns in the two countries: the educational inequalities concerning giving birth to a LBW child occurred in different levels of education. In the UK the dividing line was between high-school diploma and higher education, while in France the real difference occurred among the lowest level of education and high-school diploma. According to the authors, smoking during pregnancy could be an explanatory factor behind this phenomenon.

Several studies have also identified smoking as one of the most important risk factors. Souza et al [32] found that smoking at late pregnancy increased the risk for LBW (OR = 2.04, 95% CI: 1.60–2.59), Lamm et al [14] attributed a higher OR (OR = 2.36, 95% CI: 2.34–2.38) to smoking during pregnancy. Millar et al’s [33] results show, that maternal education and smoking during pregnancy have independent effects on small gestational age (SGA), after controlling for other risk factors. However, van den Berg et al’s path analysis showed that maternal cigarette smoking and maternal height explained the association of maternal education and LBW [19].

Several studies also pointed out the impact of depression during pregnancy on LBW. Ghimire et al [34] summarized the results of 23 studies in their meta-analysis, and they have come to the conclusion that the relative risk of LBW was 1.86 (95% CI: 1.32–2.62) in case of depression during pregnancy. In a prospective study in China, Li et al [35] also concluded that antenatal depression was associated with an increased risk for low birthweight (OR = 2.05, 95% CI: 1.12–4.64). Jarde et al’s meta-analysis [36] based on 8 studies showed that even untreated depression was associated with significantly increased risks of LBW (OR = 1.96; 95% CI: 1.24–3.10).

Birth weight can be also related to ethnicity. Based on the UK Millennium Cohort Study (MCS), [21] Indian, Pakistani, Bangladeshi, Black Caribbean and Black African infants were more likely to be low birthweight compared to White infants. Burris and Hacker [37] reported that in the USA black infants are disproportionately more likely to be born with LBW. Diabelková et al [38] pointed out that Roma mothers in Slovakia had higher risk of having LBW infants, while Hajdu et al [39] as well as Balázs et al [40] concluded similar results in Hungary.

In our analysis we also aimed to examine the effect of smoking, as a proxy for pregnancy related risk behaviour; antenatal depression, as a proxy for the psychological state of the mother during pregnancy, and Roma ethnic background, as an additional SES factor behind the influence of the maternal educational attainment to LBW.

## Materials and methods

### Study design and sample

Source of data is the Cohort ‘18 –Growing Up in Hungary national, longitudinal, birth cohort survey. The survey was launched in 2017 by the Hungarian Demographic Research Institute (HDRI) of the HCSO. The purpose of the survey is to examine the growth and development of almost 9000 children born between spring 2018 and spring 2019, from foetal age, over a longer time interval [41–43]. The main areas of data collection comprise demographics, social background, health and development.

The HDRI HCSO complied with all statistical and data protection laws currently in force in Hungary when managing the data collection of the Cohort ’18 longitudinal birth cohort study (“Act CXII of 2011 on the Right of Informational Self-Determination and on Freedom of Information”; “Regulation (EU) 2016/679 of the European Parliament and of the Council of 27 April 2016 on the protection of natural persons with regard to the processing of personal data and on the free movement of such data”; Directive 95/46/EC /”General Data Protection Regulation”). The methodology of the research was also in line with the Declaration of Helsinki and the Code of Professional Ethics of Hungarian psychologists as well. The reference number of Ethical Approval of the Ethical Committee for Cohort ‘18 Growing Up in Hungary Study is 2022/1, the date of approval is November 15, 2022.

The birth cohort is based on a representative countrywide sample. A complex multi-stage sampling procedure was used, with the primary sampling unit being the territorial health visitor district. Due to the very high coverage of the Hungarian prenatal care system by health visitors—there are approximately 4000 health visitor districts and 98% of women are seeking this service—and the relatively low rate of late foetal mortality, the sample covers the population of children born between spring 2018 and spring 2019 in Hungary almost fully. HDRI selected randomly 628 health visitor districts based on the expected number of live-births in each given health visitor district (based on HCSO live birth register data from 2015, 2016 and 2017), the geographical location (Budapest; Budapest agglomeration; large city townships from Hungary; small and medium sized geographical districts) and the estimated average social status of each health visitor district (calculated from 11 relevant indicators from the yearly reports of health visitors on the proportion of pregnant women requiring enhanced care due to environmental reasons, the proportion of perceived child neglect and child abuse cases, for example). The sample design also took into account the estimated response rates: 62 to 80% by type of settlement [44, 45]. All of the pregnant women whose due date of delivery fell between 1 April 2018 and 30 April 2019 in the selected health visitor districts were included in the sample. The size of the target population is around 90,000 and the number of the final birth cohort sample is 8600.

The planned timeframe for interviewing the expectant mothers in the first data collection wave was during weeks 28–31 of pregnancy, although the health visitor could deviate from this in certain cases. 8287 women answered the questionnaire in the prenatal wave. The second wave data were collected when the children were 6-month old, 8241 women answered the questionnaire (for 383 mothers this being the first interview). The health visitors were the interviewers in the first two waves of the survey after undergoing special training. The sample was adjusted by cell weighting procedure, according to maternal educational attainment, parity, official marital status and the mother’s age at birth (based on HCSO vital statistics and population event statistics), and also according to the economic development of the place of residence of the women [43]. The dropout rate between the pregnancy survey and the 6-month data collection (4.3%) was adjusted by weighting [46].

In the present study, we analyse the responses of those women for whom we have both pregnancy and birth-related data, who did not give birth to twins and have no missing data in predictor variable. The weighted number of cases is n = 8185.

### Measurements

The dependent variable is the birth weight of the child as is reported by the mother in the 6-month old face-to-face questionnaire. It is recoded into a dichotomous variable where the value “1” means that the mother gave birth to an LBW (<2500g) child and value “0” means that the mother gave birth to a child with 2500+ grams.

The predictor variable is the women’ educational attainment at her 7^th^ month of pregnancy reported by the mother in the prenatal and 6-month-old face-to-face questionnaire and measured by a five-value categorical variable: 1. less than eight years of schooling (corresponding to ISCED 97: 0, 1 categories); 2. eight years of schooling (ISCED 97: 2); 3. vocational education (ISCED 97: 3C); 4. secondary education (ISCED 97: 3, 4); and 5. higher education (ISCED 97: 5, 6).

There are three groups of adjustment variables in our analysis. (1) Variables indicating other socioeconomic status in addition to maternal educational attainment are the household income (divided into net monthly equivalence household income quintiles, based on a square-root equivalence scale, in accordance with OECD practice, calculated on the total sample [47]), the self-reported ethnicity (recoded into three categories: non—Roma, Roma, no answer) and the type of region of residence of women by GDP measured in 2018 (three categories: Central Hungary: the most developed NUTS2 region of Hungary; developed NUTS2 counties of Hungary; less developed NUTS2 counties of Hungary). Questions on both household income and ethnicity were self-reported. The missing values of equivalised household income (n = 1195) was imputed by the median value of the continuous equivalised household income by subjective household income categories (“In your opinion, how does your household manage the usual expenses? with great difficulty; with difficulty; with some difficulty; fairly easily; easily; very easily”). (2). Health behaviour of the women during pregnancy was measured by smoking during pregnancy (1: any cigarette; 0: no cigarette at all) and alcohol consumption during pregnancy (1: any drink, 0: no drink at all) in the prenatal face-to-face questionnaire. (3) The psychological state of the women during pregnancy was measured by depression in the prenatal wave self-reported questionnaire. Symptoms of depression were assessed using the eight-item version (CES-D-8) of the self-reported Centre for Epidemiologic Studies—Depression questionnaire [48]. The 558 missing values for depression index were imputed by the mean values of depression index by the women’s’ answer on the question “Have you had depression diagnosed by a doctor, during your pregnancy?”, and on the pregnancy risk classification according to health and social status by the health visitors. Since the measuring instrument used does not have a Hungarian cut-off value, based on the prevalence of minor and major prenatal depression (which was estimated to be between 7 and 20% in previous foreign studies in high income countries [49] and between 8 and 20% in previous Hungarian studies [50]), we considered the top 20% to be characterized by significant depressive symptoms to maximize the sensitivity of the scale.

Control variables were the maternal and paediatric biological characteristics: parity (1: the cohort child is the first child of the women; 0: not first child), child’s sex (1: female, 0: male), maternal height (continuous variable) and age of the mother at birth (4 groups: 14–24 years, 25–29 years, 30–34 years and 35–49 years) reported by the women in the prenatal and 6-month old face-to-face questionnaires.

### Statistical analysis plan

Our research interest is to examine whether LBW in Hungary is determined by maternal educational attainment. We tested this relationship by logistic regression analysis. We were also interested in testing if the relationship between maternal educational attainment and the probability of giving birth to an LBW child is still existing after adjusting for other maternal SES variables (household income, Roma ethnic background, living is less developed NUTS2 counties of Hungary); health behaviour during pregnancy (smoking and alcohol consumption); and psychological state of the women during pregnancy (depression). We estimated the crude effect and the adjusted effect of maternal educational attainment on giving birth to an LBW child. We kept the biological characteristics of mothers and children in all regression models under control. Both odds ratios and predicted probabilities are reported.

Before estimating the regression coefficients and calculating the predicted probabilities, the relationship between the dependent variable and adjusting variables, and as well between the predictor variable and adjusting variables were checked by bivariate analyses. The significant differences in the proportion of women giving birth to an LBW child by covariates were tested by Pearson Chi square, Welch coefficients and adjusted residuals.

All multivariate analyses were performed in STATA version 14 with margins and atmeans commands.

## Results

### Descriptive statistics

5.9% of women giving birth to a single child between April 2018 and April 2019 in Hungary gave birth to an LBW child (N = 8185). 52.2% of children were girls and 46.3% of children were first child of the mother (N = 8185). The mean age of the women at birth was 29.8 years (SD = 6.1 years, N = 8185, Median = 30 years), and the mean maternal height before pregnancy was 1.65 meter (SD = 0.07 meter, N = 8185; Median = 1.65 meter).

One third of the women had higher education (34.5%), another one third had secondary education (33.9%), while 11.5% had vocational education. One fifth of women in total was low educated: 17.2% had 8 years of schooling, while 2.9% had less than 8 years of schooling (N = 8185).

The mean equivalised household income was 199.2 thousand HUF (SD = 137.2 thousand HUF, N = 8185, Median = 171.8 thousand HUF). However, we used the household income quintiles in our analysis, the quintiles having cut off points as (1) 97.6 thousand HUF; (2) 146.0 thousand HUF; (3) 203.0 thousand HUF; (4) 273.7 thousand HUF. 7.5% of women identified themselves with Roma ethnic background; and for 5.3% of women we do not have information on ethnic background (N = 8185). 39.2% of women lived in less developed NUTS2 counties of Hungary, while 60.1% lived in Central Hungary (most developed region of Hungary) and more developed NUTS2 counties (N = 8127).

22.3% of women smoked during pregnancy at least occasionally (N = 8185); 13.4% of women drank alcohol during pregnancy with some frequency (N = 8185); and 21.3% showed the symptoms of the depression at the 7^th^ month of pregnancy (N = 8180).

### Bivariate analyses

**Fig 1:**
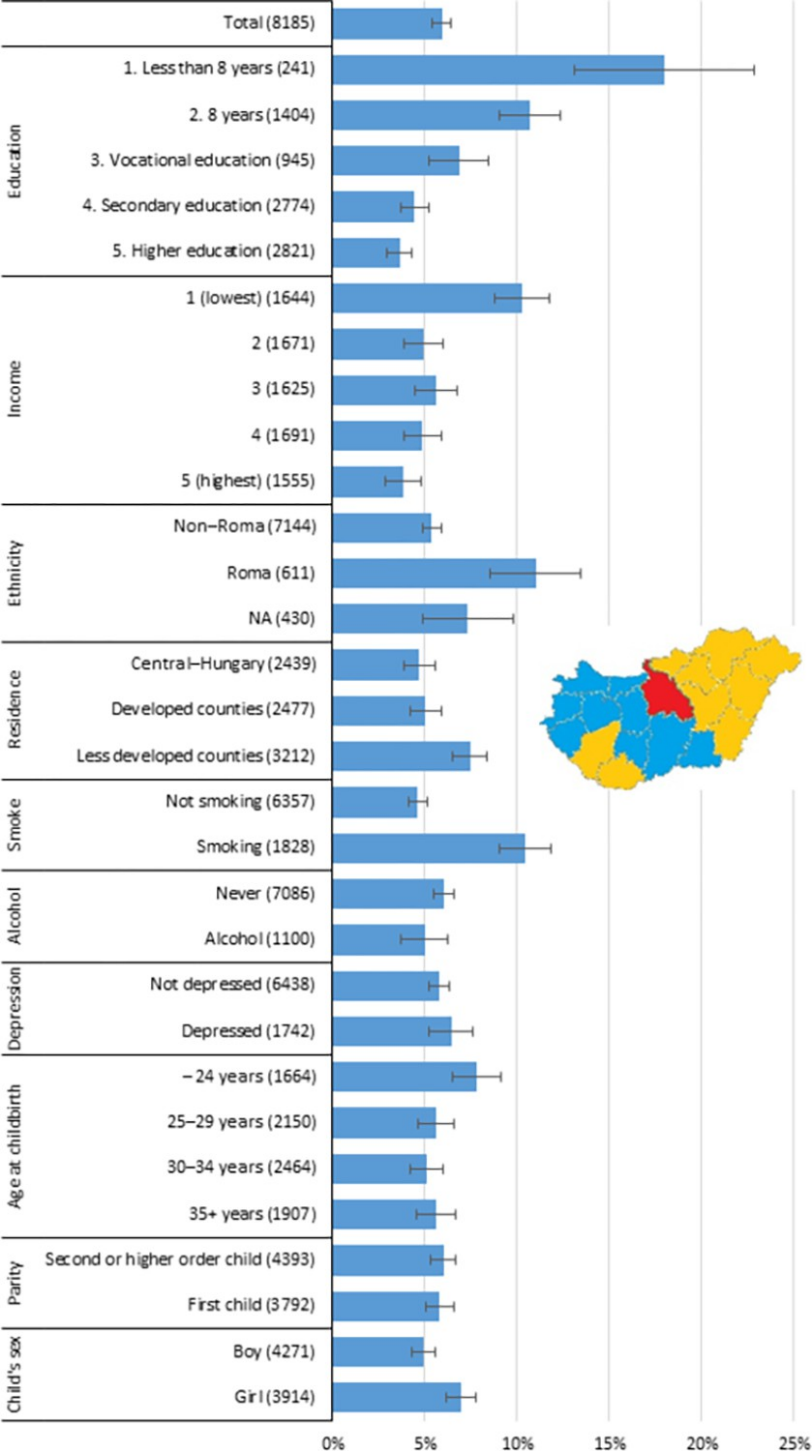
Proportion of women giving birth to a low-birthweight child, by covariates. *Note*: On the map of Hungary, the yellow areas to the east and south-west indicate the less-developed NUTS2 counties as the residence place of the mother; the blue areas of western Hungary are the developed NUTS2 counties; while the central red area is the Central Hungarian region (including the capital city, Budapest). The number of cases belonging to the given categories is indicated in parentheses. The distribution of the basic sample of the Cohort ’18 Growing Up in Hungary Hungarian longitudinal birth cohort survey can be found in the survey’s technical report [45, pp: 17. Table 2.]. Education: educational attainment of the pregnant women; income: equivalised household income quintiles; ethnicity: self-reported ethnic background of the mother; residence: residence place of the pregnant women; smoke: smoking during pregnancy with any frequancy; alcohol: drinking alcohol during pregnancy with any frequency; depression: the pregnant women is depressed at the 7^th^ month of pregnancy. *Source*: Cohort ‘18 –Growing Up in Hungary, Pregnancy and 6-month databases.

We reviewed the risk of LBW according to women’s main socioeconomic and behaviour characteristics, using bivariate analyses in the first step. Fig 1 shows the proportion of women giving birth to a LBW child, by covariates.

According to educational attainment, a negative slope is clearly discernible. The higher the educational attainment of the pregnant woman was, the lower the risk of having an LBW child was: 18.0% of women with less than eight years of schooling; 10.7% of women with eight years of schooling; 6.8% with vocational education; 4.4% with secondary education; and 3.6% with higher education had LBW-child.

Compared to the total sample, a significantly higher proportion of women gave birth to an LBW child if they had low educational attainment (either less than 8 years classes or completed 8 years classes); if they had the lowest household income; if they identified themselves as Roma; or if they lived in less-developed NUTS2 counties (S1 Table). The proportion of women who gave birth to an LBW child was also significantly higher among smokers, as well. The alcohol consumption during pregnancy and the depression at the 7^th^ month of pregnancy was not significantly related to the LBW-child-birth.

Seeing these educational inequalities in giving birth to LBW children, we wanted to further examine how is this relationship affected by other socioeconomic status indicators or by variables describing maternal behaviour and mental status during pregnancy? Considering that women with the lowest levels of education have almost 18% risk of having an LBW child, we assume that there must be an accumulation of the negative consequences, as there is significant correlation between education and the other covariates such as the household income, Roma ethnic minority background and the level of development of the region of residence, as well as between education and smoking, alcohol consumption and depression during pregnancy (Table 1).

**Table 1:** Relationship between maternal educational attainment and other covariates.

	Educational attainment of pregnant women					Total	Pearson Correlation, Sig. (2-tailed)
	1. Less than 8 years of schooling	2. 8 years of schooling	3. Vocational education	4. Secondary education	5. Higher education		
Equivalised household income, thousand HUF (mean, 95% CI, N)	85.1 (77.4–92.9; 241)	109.3 (105.5–113.1; 1404)	140.7 (135.6–145.8; 945)	194.7 (190.8–198.6; 2774)	277.6 (271.7–283.6; 2821)	199.2 (196.2–202.1; 8185)	0.465 (p = 0.000 N = 8185)
Proportion of Roma^+^(%, 95% CI, N)	41.6 (35.3–47.9; 241)	26.4 (24.1–28.7; 1404)	7.4 (5.7–9.1; 945)	2.0 (1.5–2.5; 2774)	0.5 (0.2–0.8; 2821)	7.5 (6.9–8.0; 8185)	-0.387 (p = 0.000 N = 8185)
Proportion of mothers living in less-developed NUTS2 counties^+^ (%, 95% CI, N)	70.5 (64.7–76.4; 237)	59.2 (56.6–61.8; 1387)	43.4 (40.2–46.5; 936)	37.5 (35.6–39.3; 2761)	27.9 (26.3–29.6; 2806)	39.5 (38.5–40.6; 8127)	-0.243 (p = 0.000 N = 8127)
Proportion of smokers^+^ (%, 95% CI, N)	64.9 (58.8–70.9; 241)	53.9 (51.3–56.5; 1404)	36.7 (33.6–39.8; 945)	16.3 (15.0–17.7; 2774)	4.1 (3.3–4.8; 2821)	22.3 (21.4–23.2; 8185)	-0.462 (p = 0.000 N = 8185)
Proportion of alcohol consumers^+^(%, 95% CI, N)	7.8 (4.4–11.2; 241)	8.5 (7.0–9.9; 1404)	9.0 (7.2–10.9; 945)	13.2 (11.9–14.4; 2774)	18.1 (16.7–19.5; 2821)	13.4 (12.7–14.2; 8185)	0.108 (p = 0.000 N = 8185)
Maternal depression during pregnancy (mean, 95% CI, N)	5.8 (5.3–6.3; 241)	5.7 (5.5–6.0; 1404)	5.1 (4.9–5.3; 945)	4.4 (4.2–4.5; 2770)	3.9 (3.8–4.0; 2820)	4.6 (4.5–4.6; 8180)	-0.204 (p = 0.000 N = 8180)

*Note*: ^+^ … among women with different levels of education; 95% CI: 95% Confidence intervals, N = number of women in the given category, p–significance level. *Source*: Cohort ‘18 –Growing Up in Hungary, Pregnancy and 6-month databases.

### Multivariate analysis

We applied logistic regression analysis to examine whether LBW in Hungary is affected by maternal educational attainment. The dependent variable is a binary variable where “1” means that the mother gave birth to an LBW child and “0” means the contrary. The regression model was built with (forced) Enter method. The first model includes only the maternal educational attainment and the control variables such as woman’s height, age group at childbirth, parity and child’s sex (Model 1). In the next steps we entered the adjusting variables: the maternal SES variables (Model 2); health behaviour related variables (Model 3); and the maternal psychological state variable (Model 4). We checked the interaction effects between maternal educational attainment and all other covariates in the respective models as well, but in all but one cases (when we checked the interaction between maternal education and depression during pregnancy on LBW, OR was 1.195, with 95% CI: 1.002–1.424) the effects were not significant. Thus we decided not to include them in the final multivariate models. Regression results including interaction effects are available from the authors upon request.

**Fig 2:**
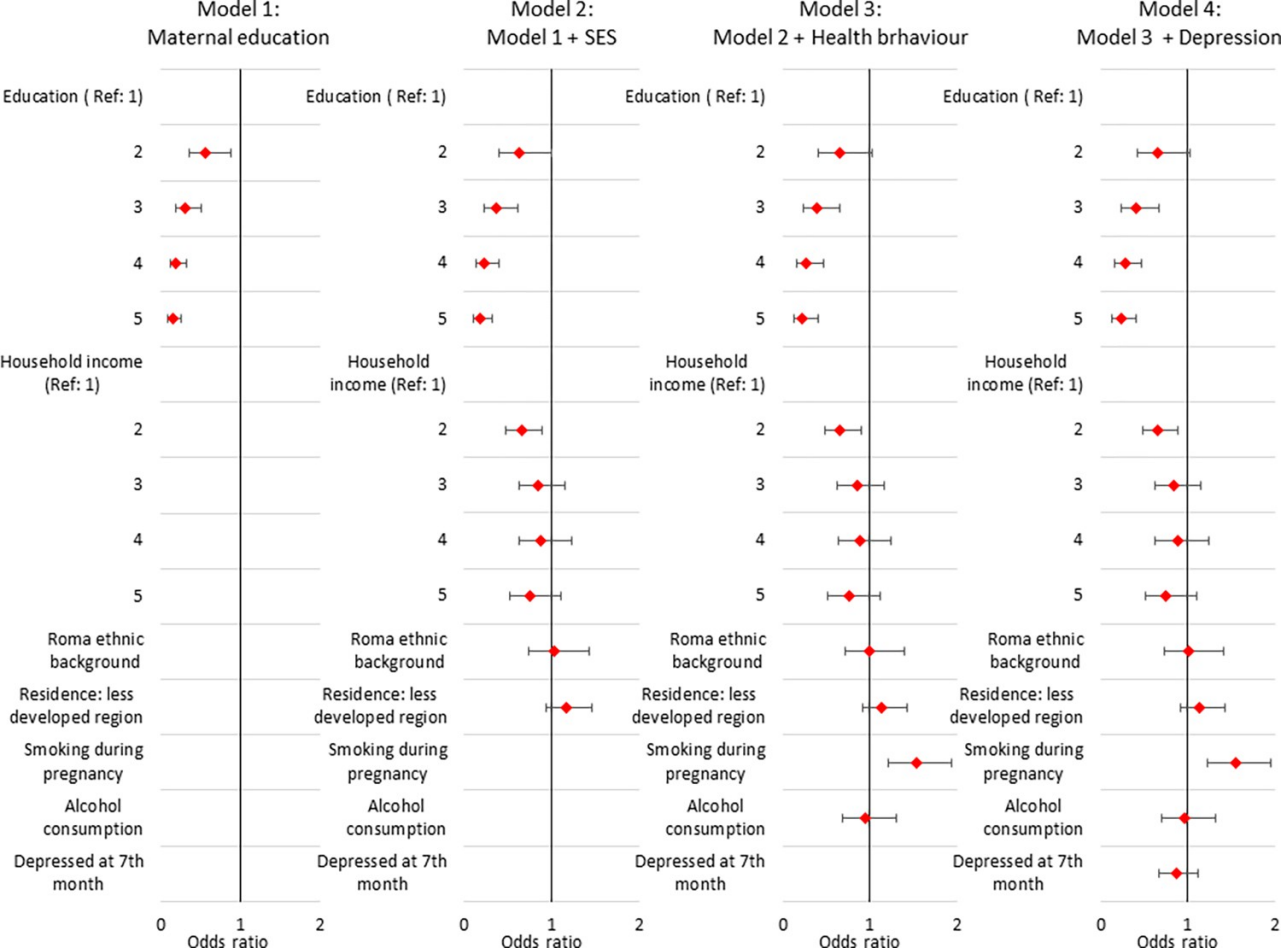
Probability of giving birth to a low-birthweight child, logistic regression analysis, odds ratios and 95% Cis. *Note*: Control variables: parity, child’s sex, mother’s height and age group. Education: educational attainment of the pregnant women: 1: less than eight years of schooling (ISCED 97: 0, 1); 2: eight years of schooling (ISCED 97: 2); 3: vocational education (ISCED 97: 3C); 4: secondary education (ISCED 97: 3, 4); 5: higher education (ISCED 97: 5, 6); household income: equivalised household income quintile groups; ethnic background Roma: self-reported Roma ethnic background of the mother; less developed NUTS2 region: residence place of the pregnant women is in the less developed NUTS2 regions of Hungary; smoking during pregnancy: smoking during pregnancy with any frequency; alcohol consumption: alcohol consumption during pregnancy with any frequency; depression or depressed at 7^th^ month of pregnancy: the pregnant women is depressed at the 7^th^ month of pregnancy; SES variables: equivalised household income quantilies, ethnic background of the mother, region of the residence place of the mother; health behaviour variables: maternal smoking and alcohol consumption during pregnancy. *Source*: Cohort ‘18 –Growing Up in Hungary, Pregnancy and 6-month databases.

The outcome of the regression model is presented in the S2 Table, while the estimated odds ratios are presented in Fig 2. The effect of maternal educational attainment on probability of LBW-child-birth is significant in all models, adjusted cumulatively for other maternal SES (Model 2), behavioural (Model 3) and psychological factors (Model 4) and controlled for maternal and paediatric biological characteristics. Compared to women with less than 8 years of schooling (level of education ISCED 97:0–1), the risk of giving birth to an LBW child decreases by 34.6% for those with 8 years of schooling level of education (ISCED 97:2); by 60.1% for those with vocational education (ISCED 97: 3C); by 72.5% for those with secondary education (ISCED 97: 3–4); and by 77.2% for those with higher education (ISCED 97: 5–6) in Model 4. Smoking during pregnancy significantly increases the risk of giving birth to an LBW child by 54.9% (OR = 1.549, 95% CI: 1.226–1.957). Being depressed at 7^th^ month of pregnancy and giving birth to an LBW child are in inverse relationship in our sample, however, the relationship is not significant (OR = 0.868, 95% CI: 0.676–1.114).

### Predicted probabilities of giving birth to low-birthweight child by maternal educational attainment

**Fig 3:**
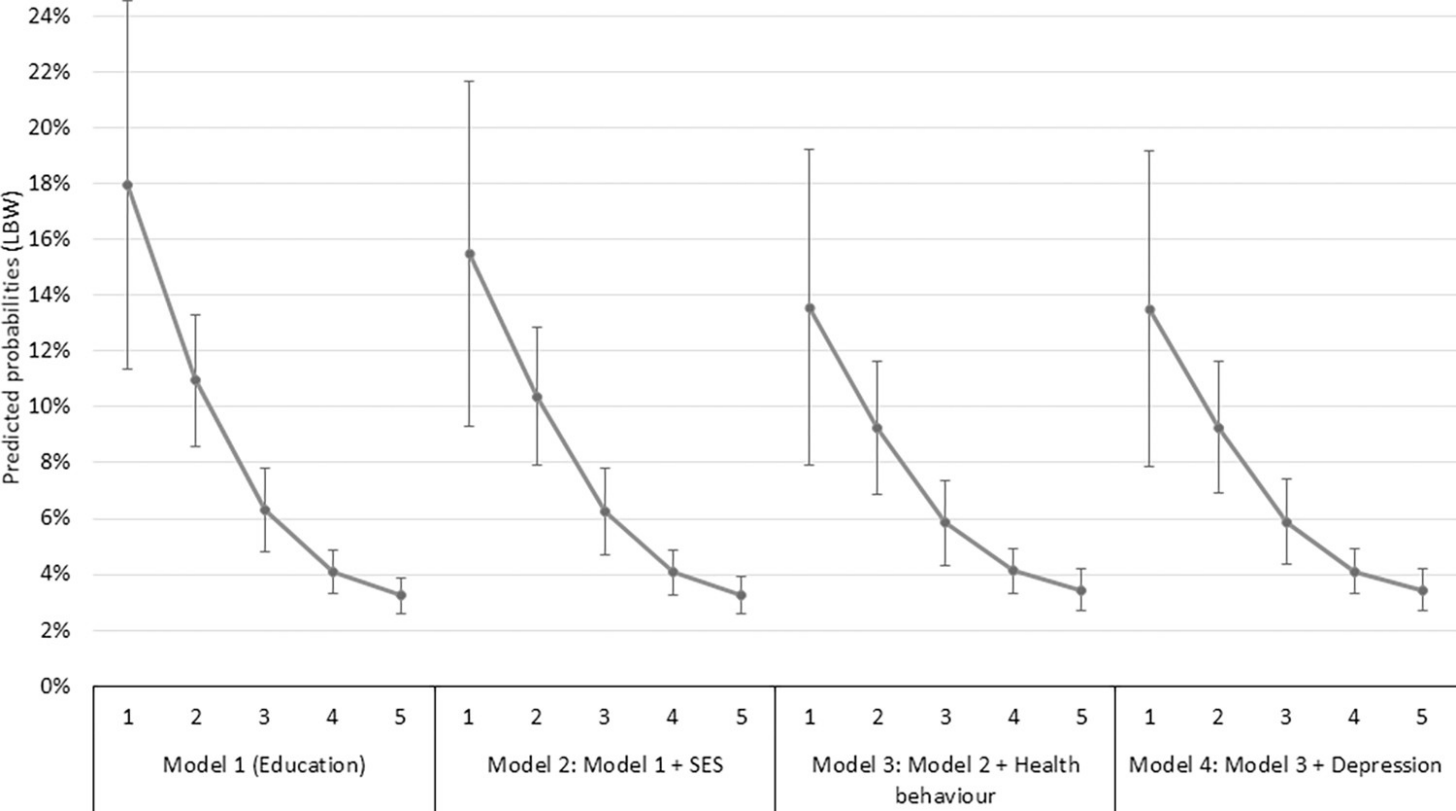
Adjusted predicted probabilities of giving birth to a low-birthweight child, by maternal educational attainment. *Note*: Binary logistic regression analysis. Control variables: parity, sex of child, height of mother, age groups of mother. Figures on x-axis stands for the maternal educational attainment: 1: less than eight years of schooling (ISCED 97: 0, 1); 2: eight years of schooling (ISCED 97: 2); 3: vocational education (ISCED 97: 3C); 4: secondary education (ISCED 97: 3, 4); 5: higher education (ISCED 97: 5, 6). See also S3 Table. SES variables: equivalised household income quantilies, ethnic background of the mother, region of the residence place of the mother; health behaviour variables: maternal smoking and alcohol consumption during pregnancy; depression: the pregnant women is depressed at the 7^th^ month of pregnancy. *Source*: Cohort ‘18 –Growing Up in Hungary, Pregnancy and 6-month databases.

We used a bivariate logistic regression analyses to estimate the predicted probability of a woman giving birth to an LBW child according to her educational attainment, adjusted for other socio-economic characteristics, health behaviour and psychological state factors, while controlling for maternal and paediatric baseline characteristics, such as parity, sex of the child, height and age of the mother (Fig 3).

In the first model, we estimated the adjusted predicted probability of giving birth to LBW child across mothers’ educational levels, controlled for the basic maternal and child demographics variables. Compared to the unadjusted value of 18% of women with the lowest level of education (with less than 8 classes) having an LBW child and 3.6% of the highest educated women (with higher education), in this first adjusted model the lowest educated women have a 17.9%, and the highest educated women have a 3.2% probability of giving birth to an LBW child (Model 1). After adjusting the model with all other SES characteristics (Model 2), we estimated that 15.5% of women with the lowest level of education and 3.3% with the highest education gave birth to an LBW child. If we further adjust the regression model for the frequency of smoking and alcohol consumption during pregnancy (Model 3), then the estimated probability of a woman with the lowest educational attainment giving birth to an LBW child is only 13.6% and 3.4% for the highest educated women. Finally, if we also adjust the regression model for the maternal depression during 7^th^ month of pregnancy (Model 4), then the estimated probability of a woman with the lowest educational attainment giving birth to an LBW child becomes 13.5% for the lowest and 3.4% for the highest educated women.

## Discussion

According to the international literature, one of the most relevant determinants of LBW is maternal social status. Different levels of SES—which is relatively well captured by educational attainment—create different opportunities and limitations for someone to be financially secure and physically and mentally healthy: to eat the right amount and quality of food, have access to quality health care, participate in pregnancy care in a timely manner, having an appropriate (e.g. pollution-free) environment both before and during pregnancy, and to avoid risk behaviours during pregnancy, e.g. smoking, alcohol and drug use [24, 26]. Our analysis confirmed that maternal educational attainment has a significant impact on the risk of LBW, as it was stated in several previous studies [12, 16, 18, 19]. Moreover, we found evidence that this relationship still persists even if we adjust it for other maternal characteristics that are closely related to educational attainment such as various maternal SES factors, as well as health behaviour during pregnancy. However, while lower household income, Roma ethnicity, smoking during pregnancy and living in a less developed region increased the probability of LBW infants in bivariate analysis, only maternal smoking remained significant risk factor of LBW beside maternal education in the multivariate regression model, when adjusted for all covariates.

Thus our results support the importance of smoking as a risk factor for LBW [14, 19, 32, 33], but we cannot confirm the role of (Roma) ethnicity or the positive effect of prenatal depression to LBW. The latter findings partly contradicts to the international results as depression has been shown in the literature to increase the risk of LBW, although there are some findings to the contrary. In our study depression increased the risk of LBW only at higher levels of education, while the opposite was seen at lower levels of education (the differences however were not significant)–the reasons for this should be investigated in further research.

Nevertheless, we have to emphasize that even after adjusting for maternal SES, behavioural and psychological factors, LBW inequality by maternal educational attainment persists. All other things being equal, poorly educated women are more likely to have an LBW child than are their better-educated peers: 13.5% versus 3.4%.

**Fig 4:**
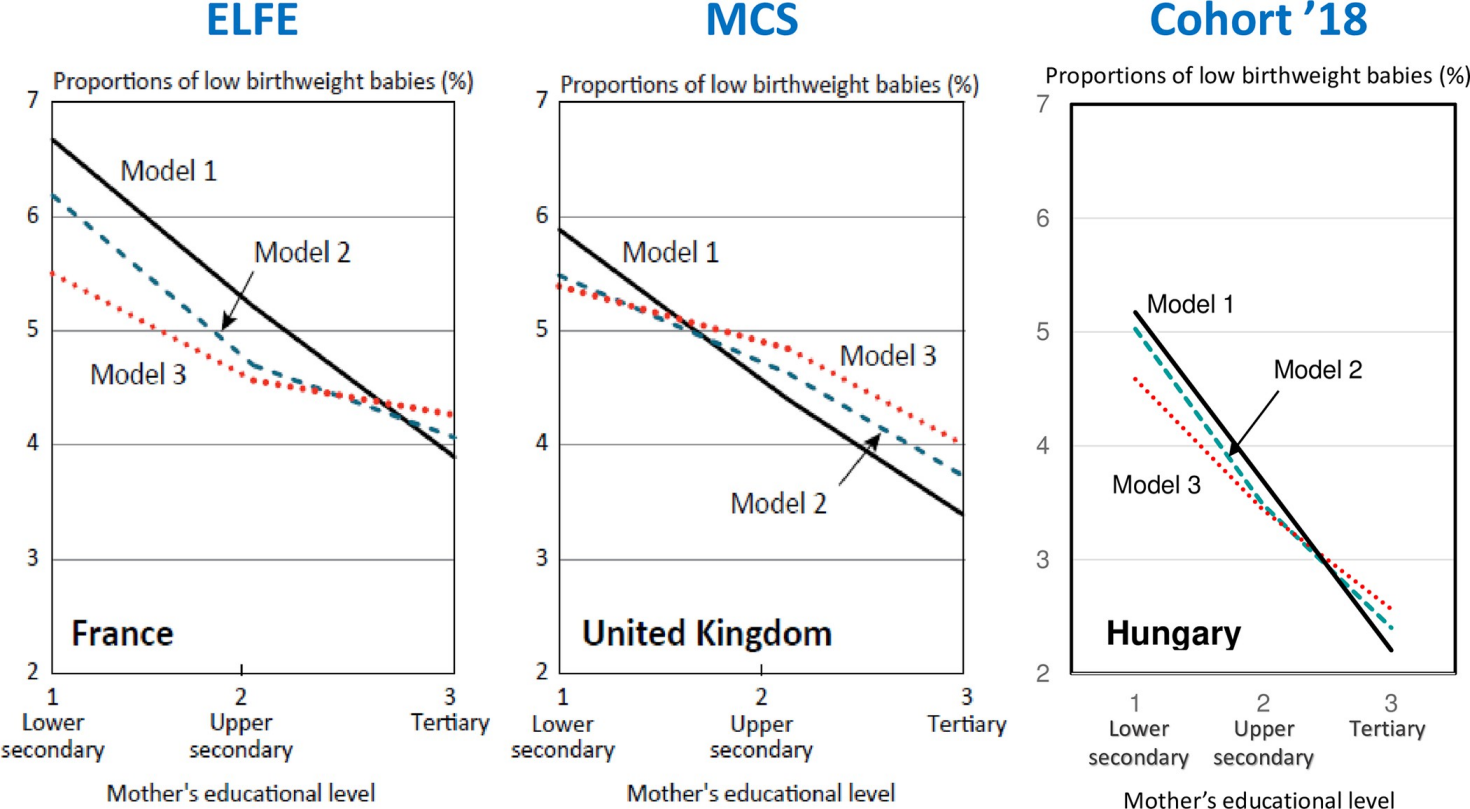
Risk of having a low-birthweight child by educational attainment of the mother: Comparison of France, UK and Hungary–logistic regression analysis, predicted probabilities, adjusted for parity, child’s sex and mother’s height. *Note*: Model 1: baby’s sex, whether the baby is a first child, the mother’s age group, the height of the mother (meter). Educational attainment of the pregnant women: lower secondary (ISCED 97: 0, 1, 2, 3C); upper secondary (ISCED 97: 3, 4); tertiary (ISCED 97: 5, 6). Model 2: Model 1 + equivalised household income quantiles. Model 3: Model 2 + drinking and smoking during pregnancy. Only singleton births at more than 33 weeks’ gestation to mothers aged 18 or over were included in all three surveys for this comparison. *Source*: France: French Longitudinal Study of Children (ELFE) survey (2011); United Kingdom: Millennium Cohort Study (MCS) survey (2001) ([11]: Fig 3); Hungary: Cohort ‘18 –Growing Up in Hungary (2018–2019). See S4 Table for marginal effects of the adjusted predicted probabilities and their 95% confidence interval, for Hungarian data.

Replicating Panico et al’s analysis [26] for the Hungarian data, we found that—as in France—there is a difference in the risk of giving birth to a LBW child between those with lower secondary education or less (vocational education) and upper secondary education, rather than between those with upper secondary and tertiary education (Fig 4). Thus, we can observe how the group of mothers with secondary education is somewhat ‘protected’ against the risk of low-birthweight in Hungary.

## Strengths and limitations

Our study is based on a nationally representative sample, so the results are generalizable.

Our analysis confirmed that mother’s educational attainment has an impact on the risk of low birthweight: the higher the maternal educational attainment, the lower the risk of giving birth to an LBW-child. However, our analysis went behind this fact as it led to results that are more detailed. We can assume that the association between mother’s educational attainment and LBW is mitigated by other SES factors and maternal health behaviour during pregnancy. Inequality in LBW by maternal educational attainment is decreasing when adjusted for maternal SES, health behaviour and psychological state factors. At the same time, even if this buffering effect exists, inequality in LBW by maternal educational level persists after adjusting for other maternal SES, behaviour and psychological factors.

One of the limitations of our analysis is that, due to the specificity of the data collection, children born before the seventh month of pregnancy were not included in the sample, so we could not analyse the data of those born with extremely low weight. In future analyses, it would also be worth noting the distinction between the two types of low-birth weight infants: preterm infants and those born with intrauterine developmental delay.

## Conclusion

The risk factors of LBW are significantly interrelated and thus the negative consequences are concentrated in families that face several disadvantages [51, 52], as our results confirmed. However, the poorer health of children at birth with low-SES parents may be caused not by worse genetic endowments, but by the circumstances of gestation, pregnancy and birth [53]. Lower-SES mothers not only have greater barriers to healthcare facilities and a healthy environment, but also tend to indulge in more risk behaviour during pregnancy [54].

We believe that it is the responsibility of government, professional groups, schools and the mass media to focus attention on LBW: focusing on the causes and consequences, and raising awareness of possible ways of prevention. Inevitably, great importance must be attached to educating the population on pregnancy generally, and on low birthweight in particular; and to ensuring the availability of comprehensive family planning services, especially for low-income women and adolescents. There are several risk factors that can be identified by health visitors and obstetricians well before conception. For example, if the woman suffers from chronic illness, smokes, consumes alcohol or abuses substances; if she is underweight for her height or has poor nutrition; if she suffers from specific infections; if she is very young or relatively old (under 17 or over 34); if there is the possibility of a very short interval between pregnancies or high parity. Early identification of these groups and early protection and provision of evidence-based information can reduce the risk of LBW infants.

Thus, affordable, accessible and appropriate health care is critical to preventing and treating LBW especially among women with low socioeconomic status. While among women with high socioeconomic status the depressive symptoms have to be care of. Evidence-informed interventions to prevent LBW should be implemented not only at the country or community level, but also at the individual level, through pre-pregnancy and antenatal care interventions.

## Supporting information

S1 Fig The share of newborns with low birthweight in selected European countries, 1980–2018. *Note*: Source: OECD Health Statistics 2020, Eurostat Database and national source for Croatia and Cyprus, 1980–2018.
(TIF)

S2 Fig The share of newborns with low birthweight, by mother’s educational attainment, Hungary, 1990–2018. *Note*: Source: Hungarian Central Statistics Office Vital Statistics, 1990–2018, own calculation.
(TIF)

S1 Table Proportion of women giving birth to a low-birthweight child, by covariates. Crosstable analysis, row %, Chi-square coefficient, Sig. 2-tailed, and Adjusted residuals. *Note*: Source: Cohort ‘18 –Growing Up in Hungary (2018–2019), Pregnancy and 6-month databases, own calculation.
(DOCX)

S2 Table Estimated probability of giving birth to a low-birthweight child, by educational attainment. Logistic regression analysis, Exp(B) and 95% confidence intervals, significance level. *Note*: Logistic regression analysis, N = 8115. Wald statistics’ significance are presented in the table. Control variables: mother’s age at birth, parity, sex of child, height of mother. Collinearity statistics: Tolerance [0.751–1.000], VIF [1.000–1.330]. Source: Cohort ‘18 –Growing Up in Hungary (2018–2019), Pregnancy and 6-month databases, own calculation.
(DOCX)

S3 Table Estimated marginal means of probability of giving birth to a low-birthweight child, by maternal educational attainment, by models. Margins, standard errors, z values, P>|z| and 95% confidence intervals. *Note*: Covariates appearing in the model are evaluated at the following values: Parity = 1.81, Child’s sex = 0.48, Mother’s height, m = 1.65, Mother’s age group: 14–24 years = 0.20; 25–29 years = 0.26; 30–39 years = 0.30; 40–49 years = 0.23. SES: equivalised household income quintiles. Education: educational attainment of the pregnant women; SES variables: equivalised household income quantilies, ethnic background of the mother, region of the residence place of the mother; smoke, alcohol consumption: maternal smoking and alcohol consumption during pregnancy; depression: the pregnant women is depressed at the 7th month of pregnancy. Source: Cohort ‘18 –Growing Up in Hungary (2018–2019), own calculation.
(DOCX)

S4 Table Estimated probability of giving birth to a low-birthweight child, by educational attainment. Predicted probabilities, adjusted predictions, Margins, 95% CI, Hungary. *Note*: Binary logistic regression analysis, unadjusted and adjusted predicted probabilities/margins. Educational attainment of the pregnant women: lower secondary (ISCED 97: 0, 1, 2, 3C); upper secondary (ISCED 97: 3, 4); tertiary (ISCED 97: 5, 6). Model 1: baby’s sex, whether the baby is a first child, the mother’s age, the height of the mother (meter). Model 2: Model 1 + equivalised household income per capita quantiles. Model 3: Model 2 + maternal drinking and smoking during pregnancy. Only singleton births at more than 33 weeks’ gestation to mothers aged 18 or over were included in all three surveys for these analyses. Source: Cohort ‘18 –Growing Up in Hungary (2018–2019), own calculation.
(DOCX)
