# Supplementary figures and images for: Socio-economic differences among low-birthweight infants in Hungary. Results of the Cohort ‘18 –Growing Up in Hungary birth cohort study
Source: PLoS One. 2023 Sep 1;18(9):e0291117. doi: 10.1371/journal.pone.0291117 (PMC10473525; doi:10.1371/journal.pone.0291117)

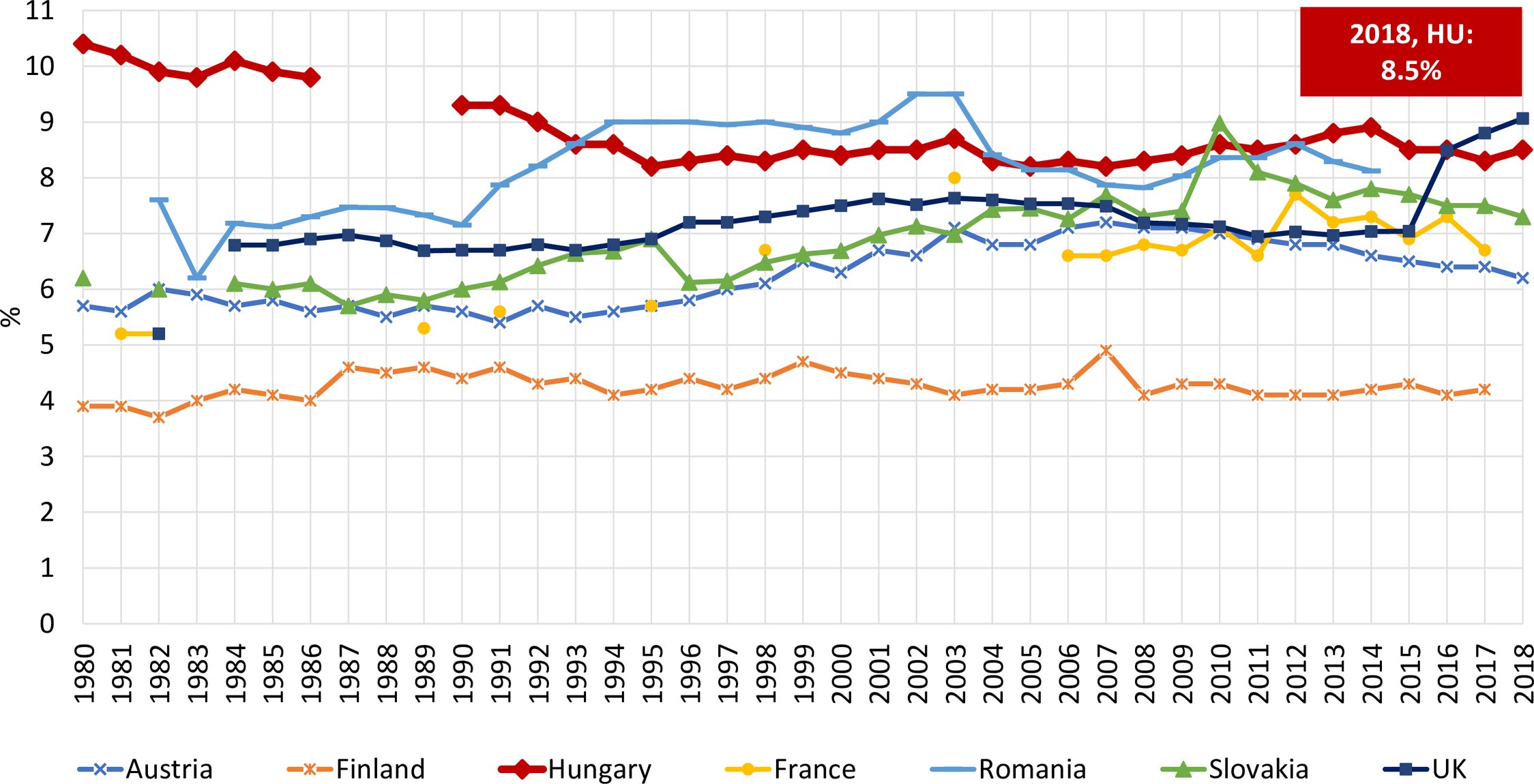

Supplement: S1 Fig — Note: Source: OECD Health Statistics 2020, Eurostat Database and national source for Croatia and Cyprus, 1980–2018. (TIF) [file pone.0291117.s001.tif]

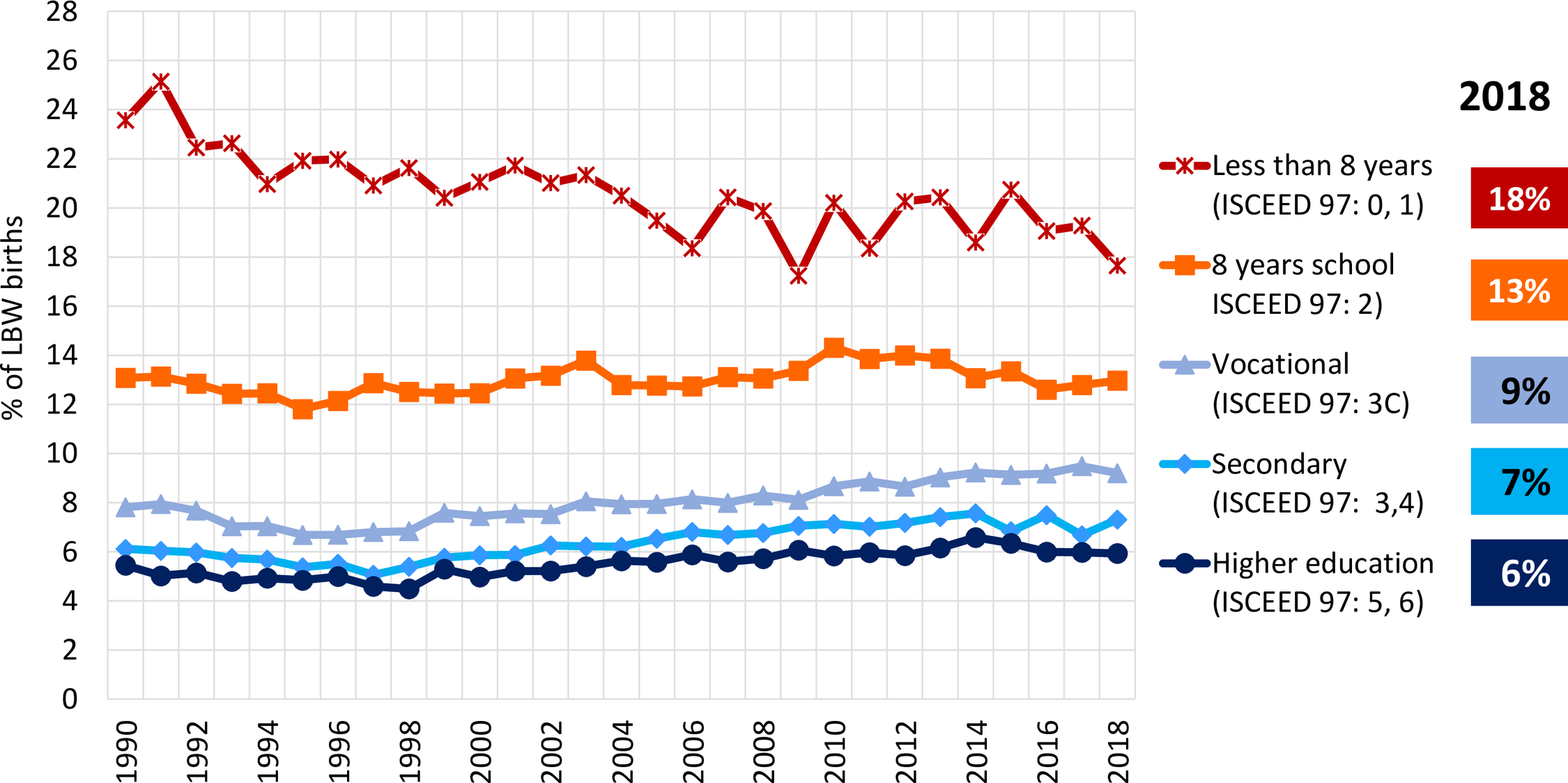

Supplement: S2 Fig — Note: Source: Hungarian Central Statistics Office Vital Statistics, 1990–2018, own calculation. (TIF) [file pone.0291117.s002.tif]
