# Supplementary material for: Socio-economic differences among low-birthweight infants in Hungary. Results of the Cohort ‘18 –Growing Up in Hungary birth cohort study
Source: PLoS One. 2023 Sep 1;18(9):e0291117. doi: 10.1371/journal.pone.0291117 (PMC10473525; doi:10.1371/journal.pone.0291117)
Supplement: S1 Table — Crosstable analysis, row %, Chi-square coefficient, Sig. 2-tailed, and Adjusted residuals. Note: Source: Cohort ‘18 –Growing Up in Hungary (2018–2019), Pregnancy and 6-month databases, own calculation. (DOCX) [file pone.0291117.s003.docx]

**S1 Table. Proportion of women giving birth to a low-birthweight child, by covariates. Crosstable analysis, row %, Chi-square coefficient, Sig. 2-tailed, and Adjusted residuals.**

|  |  |  | **Share of mothers giving birth to a low-birthweight child** | | | **Phi/Cramer's V Symmetric Measure of Association; Approx. Sign.** |
| --- | --- | --- | --- | --- | --- | --- |
|  |  |  | **>=2500g** | **<2500g** | **Total** |  |
| **Educational attainment of the pregnant women** | **1. < 8 years of classes** | Row % | 82.0% | 18.0% | 241 | 0.140;  0.000 |
|  |  | Adjusted Residual | -8.06 | 8.06 |  |  |
|  | **2. 8 years of classes** | Row % | 89.3% | 10.7% | 1404 |  |
|  |  | Adjusted Residual | -8.36 | 8.36 |  |  |
|  | **3. Vocational education** | Row % | 93.2% | 6.8% | 945 |  |
|  |  | Adjusted Residual | -1.29 | 1.29 |  |  |
|  | **4. Secondary education** | Row % | 95.6% | 4.4% | 2774 |  |
|  |  | Adjusted Residual | 4.02 | -4.02 |  |  |
|  | **5. Higher education** | Row % | 96.4% | 3.6% | 2821 |  |
|  |  | Adjusted Residual | 6.36 | -6.36 |  |  |
|  | **Total** | Row % | 94.1% | 5.9% | 8185 |  |
| **Equivalised household income quantiles** | **1 (lowest)** | Row % | 89.7% | 10.3% | 1644 | 0.096;  0.000 |
|  |  | Adjusted Residual | -8.42 | 8.42 |  |  |
|  | **2** | Row % | 95.1% | 4.9% | 1671 |  |
|  |  | Adjusted Residual | 1.91 | -1.91 |  |  |
|  | **3** | Row % | 94.4% | 5.6% | 1625 |  |
|  |  | Adjusted Residual | 0.60 | -0.60 |  |  |
|  | **4** | Row % | 95.1% | 4.9% | 1691 |  |
|  |  | Adjusted Residual | 2.06 | -2.06 |  |  |
|  | **5 (highest)** | Row % | 96.2% | 3.8% | 1555 |  |
|  |  | Adjusted Residual | 3.89 | -3.89 |  |  |
|  | **Total** | Row % | 94.1% | 5.9% | 8185 |  |
| **Ethnic background of the mother** | **Non-Roma** | Row % | 94.6% | 5.4% | 7144 | 0.064;  0.000 |
|  |  | Adjusted Residual | 5.22 | -5.22 |  |  |
|  | **Roma** | Row % | 89.0% | 11.0% | 611 |  |
|  |  | Adjusted Residual | -5.53 | 5.53 |  |  |
|  | **No answer** | Row % | 92.7% | 7.3% | 430 |  |
|  |  | Adjusted Residual | -1.27 | 1.27 |  |  |
|  | **Total** | Row % | 94.1% | 5.9% | 8185 |  |
| **Region of residence of the pregnant women** | **Central-Hungary** | Row % | 95.3% | 4.7% | 2439 | 0.054;  0.000 |
|  |  | Adjusted Residual | 3.01 | -3.01 |  |  |
|  | **Developed NUTS2 counties** | Row % | 94.9% | 5.1% | 2477 |  |
|  |  | Adjusted Residual | 2.10 | -2.10 |  |  |
|  | **Less developed NUTS2 counties** | Row % | 92.6% | 7.4% | 3212 |  |
|  |  | Adjusted Residual | -4.80 | 4.80 |  |  |
|  | **Total** | Row % | 94.1% | 5.9% | 8127 |  |
| **Smoking during pregnancy** | **No** | Row % | 95.4% | 4.6% | 6357 | 0.102;  0.000 |
|  |  | Adjusted Residual | 9.24 | -9.24 |  |  |
|  | **Yes** | Row % | 89.6% | 10.4% | 1828 |  |
|  |  | Adjusted Residual | -9.24 | 9.24 |  |  |
|  | **Total** | Row % | 94.1% | 5.9% | 8185 |  |
| **Alcohol consumption during pregnancy** | **No** | Row % | 93.9% | 6.1% | 7086 | -0.016;  0.159 |
|  |  | Adjusted Residual | -1.41 | 1.41 |  |  |
|  | **Yes** | Row % | 95.0% | 5.0% | 1100 |  |
|  |  | Adjusted Residual | 1.41 | -1.41 |  |  |
|  | **Total** | Row % | 94.1% | 5.9% | 8185 |  |
| **Depression at 7th month of pregnancy** | **Not depressed** | Row % | 94.2% | 5.8% | 6438 | 0.011  0.313 |
|  |  | Adjusted Residual | 1.01 | -1.01 |  |  |
|  | **Depressed (Top 20%)** | Row % | 93.6% | 6.4% | 1742 |  |
|  |  | Adjusted Residual | -1.01 | 1.01 |  |  |
|  | **Total** | Row % | 94.1% | 5.9% | 8180 |  |

*S1 Table Note*: Source: Cohort ’18 – Growing Up in Hungary (2018–2019), Pregnancy and 6-month databases, own calculation.
