# Supplementary material for: Socio-economic differences among low-birthweight infants in Hungary. Results of the Cohort ‘18 –Growing Up in Hungary birth cohort study
Source: PLoS One. 2023 Sep 1;18(9):e0291117. doi: 10.1371/journal.pone.0291117 (PMC10473525; doi:10.1371/journal.pone.0291117)
Supplement: S2 Table — Logistic regression analysis, Exp(B) and 95% confidence intervals, significance level. Note: Logistic regression analysis, N = 8115. Wald statistics’ significance are presented in the table. Control variables: mother’s age at birth, parity, sex of child, height of mother. Collinearity statistics: Tolerance [0.751–1.000], VIF [1.000–1.330]. Source: Cohort ‘18 –Growing Up in Hungary (2018–2019), Pregnancy and 6-month databases, own calculation. (DOCX) [file pone.0291117.s004.docx]

**S2 Table. Estimated probability of giving birth to a LBW child, by maternal educational attainment. Logistic regression analysis, Exp(B) and 95% CI, significance level**

| **Variables in equation** | **Model 1** | **Model 2** | **Model 3** | **Model 4** |
| --- | --- | --- | --- | --- |
| **Educational attainment of the pregnant women** | 0.641  (0.584-0.700)  Sig.= 0.000 | 0.662  (0.596-0.737)  Sig.= 0.000 | 0.704  (0.629-0.787)  Sig.= 0.000 | 0.703  (0.628-0.786)  Sig.= 0.000 |
| **Equivalised household income quantiles** |  | 0.972  (0.893-1.059)  Sig.= 0.520 | 0.975  (0.896-1.062)  Sig.= 0.568 | 0.972  (0.892-1.059)  Sig.= 0.513 |
| **Ethnic background of the mother (Ref: Non-Roma)** | | | | |
| **Roma** |  | 1.048  (0.771-1.424)  Sig.= 0.764 | 1.020  (0.750-1.386)  Sig.= 0.900 | 1.029  (0.757-1.399)  Sig.= 0.856 |
| **No answer** |  | 1.206  (0.818-1.776)  Sig.= 0.344 | 1.200  (0.814-1.770)  Sig.= 0.356 | 1.197  (0.812-1.765)  Sig.= 0.364 |
| **Residence place of the pregnant women (Ref: Central Hungary and developed NUTS2 counties** | | | | |
| **Less developed NUTS2 counties** |  | 1.178  (0.964-1.439)  Sig.= 0.110 | 1.148  (0.938-1.405)  Sig.= 0.182 | 1.148  (0.937-1.405)  Sig.= 0.182 |
| **Smoking during pregnancy** |  |  | 1.518  (1.214-1.898)  Sig.= 0.000 | 1.530  (1.223-1.915)  Sig.= 0.000 |
| **Alcohol consumption during pregnancy** |  |  | 0.965  (0.717-1.300)  Sig.= 0.817 | 0.972  (0.722-1.309)  Sig.= 0.852 |
| **Depressed at 7^th^ month of pregnancy** |  |  |  | 0.879  (0.699-1.104)  Sig.= 0.267 |
| **Constant** | 523.883  Sig.= 0.000 | 363.988  Sig.= 0.000 | 230.136  Sig.= 0.000 | 249.134  Sig.= 0.000 |
| **Initial -2 Log Likelihood:** | 3636.970 |  |  |  |
| **Model -2 Log Likelihood:** | 3431.028 | 3426.616 | 3413.436 | 3412.181 |
| **Model Chi-Square Sig.** | p=0.000 | p=0.000 | p=0.000 | p=0.000 |
| **Step Chi-Square Sig** | p=0.000 | p=0.353 | p=0.001 | p=0.263 |
| **Nagelkerke R^2^** | 6.94% | 7.08% | 7.52% | 7.56% |
| **McFadden’s R^2^** | 5.66% | 5.78% | 6.15% | 6.18% |
| **Hosmer and Lemeshow Chi-Square Test Sig.** | p=0.951 | p=0.555 | p=0.520 | p=0.472 |
| **Overall percentage** | 94.1% | 94.1% | 94.1% | 94.1% |

### *S2 Table Note*: Logistic regression analysis, N=8115. Wald statistics’ significance are presented in the table. Control variables: mother’s age at birth, parity, sex of child, height of mother. Collinearity statistics: Tolerance [0.767–1.000], VIF [1.000–1.303]. Source: Cohort ’18 – Growing Up in Hungary (2018–2019), Pregnancy and 6-month databases, own calculation.
