# Supplementary material for: Socio-economic differences among low-birthweight infants in Hungary. Results of the Cohort ‘18 –Growing Up in Hungary birth cohort study
Source: PLoS One. 2023 Sep 1;18(9):e0291117. doi: 10.1371/journal.pone.0291117 (PMC10473525; doi:10.1371/journal.pone.0291117)
Supplement: S3 Table — Margins, standard errors, z values, P>|z| and 95% confidence intervals. Note: Covariates appearing in the model are evaluated at the following values: Parity = 1.81, Child’s sex = 0.48, Mother’s height, m = 1.65, Mother’s age group: 14–24 years = 0.20; 25–29 years = 0.26; 30–39 years = 0.30; 40–49 years = 0.23. SES: equivalised household income quintiles. Education: educational attainment of the pregnant women; SES variables: equivalised household income quantilies, ethnic background of the mother, region of the residence place of the mother; smoke, alcohol consumption: maternal smoking and alcohol consumption during pregnancy; depression: the pregnant women is depressed at the 7th month of pregnancy. Source: Cohort ‘18 –Growing Up in Hungary (2018–2019), own calculation. (DOCX) [file pone.0291117.s005.docx]

**S3 Table. Estimated marginal means of probability of giving birth to a low-birthweight child, by maternal educational attainment, by regression models. Margins, standard errors, z value, P>|z| and 95% confidence intervals**

**Dependent variable: Giving birth to LBW children**

|  | **Mother’s educational level** | **Margin** | **Std. Err.** | **z** | **P>\|z\|** | **95% Confidence Interval** | |
| --- | --- | --- | --- | --- | --- | --- | --- |
|  |  |  |  |  |  | **Lower Bound** | **Upper Bound** |
| **Model 1: Education** | **1. Less than 8 years** | 0.159 | 0.020 | 7.790 | 0.000 | 0.119 | 0.199 |
|  | **2. 8 years** | 0.106 | 0.010 | 10.870 | 0.000 | 0.087 | 0.125 |
|  | **3. Vocational education** | 0.070 | 0.004 | 17.030 | 0.000 | 0.062 | 0.078 |
|  | **4. Secondary education** | 0.045 | 0.003 | 17.830 | 0.000 | 0.040 | 0.050 |
|  | **5. Higher education** | 0.029 | 0.003 | 10.860 | 0.000 | 0.024 | 0.034 |
| **Model 2: Model 1 + SES** | **1. Less than 8 years** | 0.149 | 0.022 | 6.930 | 0.000 | 0.107 | 0.192 |
|  | **2. 8 years** | 0.102 | 0.010 | 9.760 | 0.000 | 0.081 | 0.122 |
|  | **3. Vocational education** | 0.068 | 0.004 | 15.890 | 0.000 | 0.060 | 0.076 |
|  | **4. Secondary education** | 0.045 | 0.003 | 17.760 | 0.000 | 0.040 | 0.050 |
|  | **5. Higher education** | 0.029 | 0.003 | 10.440 | 0.000 | 0.024 | 0.035 |
| **Model 3: Model 2 + smoke, alcohol consumption** | **1. Less than 8 years** | 0.128 | 0.020 | 6.250 | 0.000 | 0.088 | 0.168 |
|  | **2. 8 years** | 0.091 | 0.010 | 8.920 | 0.000 | 0.071 | 0.111 |
|  | **3. Vocational education** | 0.064 | 0.004 | 14.880 | 0.000 | 0.056 | 0.073 |
|  | **4. Secondary education** | 0.045 | 0.003 | 17.740 | 0.000 | 0.040 | 0.050 |
|  | **5. Higher education** | 0.031 | 0.003 | 10.230 | 0.000 | 0.025 | 0.037 |
| **Model 4: Model 3 + Depression** | **1. Less than 8 years** | 0.128 | 0.021 | 6.250 | 0.000 | 0.088 | 0.168 |
|  | **2. 8 years** | 0.091 | 0.010 | 8.920 | 0.000 | 0.071 | 0.111 |
|  | **3. Vocational education** | 0.064 | 0.004 | 14.880 | 0.000 | 0.056 | 0.073 |
|  | **4. Secondary education** | 0.045 | 0.003 | 17.710 | 0.000 | 0.040 | 0.050 |
|  | **5. Higher education** | 0.031 | 0.003 | 10.200 | 0.000 | 0.025 | 0.037 |

*S3 Table Note:* Covariates appearing in the model are evaluated at the following values: Parity = 0.48, Child’s sex = 0.48, Mother’s height, m = 1.65, Mother’s agegroup: 14–24 years =0.20; 25–29 years=0.26; 30–39 years= 0.30; 40–49 years=0.23. SES: equivalized household income quintiles. Education: educational attainment of the pregnant women; SES variables: equivalized household income quantilies, ethnic background of the mother, region of the residence place of the mother; smoke, alcohol consumption: maternal smoking and alcohol consumption during pregnancy; depression: the pregnant women is depressed at the 7th month of pregnancy. *Source:* Cohort ’18 – Growing Up in Hungary (2018–2019), own calculation.
