# Supplementary material for: Socio-economic differences among low-birthweight infants in Hungary. Results of the Cohort ‘18 –Growing Up in Hungary birth cohort study
Source: PLoS One. 2023 Sep 1;18(9):e0291117. doi: 10.1371/journal.pone.0291117 (PMC10473525; doi:10.1371/journal.pone.0291117)
Supplement: S4 Table — Predicted probabilities, adjusted predictions, Margins, 95% CI, Hungary. Note: Binary logistic regression analysis, unadjusted and adjusted predicted probabilities/margins. Educational attainment of the pregnant women: lower secondary (ISCED 97: 0, 1, 2, 3C); upper secondary (ISCED 97: 3, 4); tertiary (ISCED 97: 5, 6). Model 1: baby’s sex, whether the baby is a first child, the mother’s age, the height of the mother (meter). Model 2: Model 1 + equivalised household income per capita quantiles. Model 3: Model 2 + maternal drinking and smoking during pregnancy. Only singleton births at more than 33 weeks’ gestation to mothers aged 18 or over were included in all three surveys for these analyses. Source: Cohort ‘18 –Growing Up in Hungary (2018–2019), own calculation. (DOCX) [file pone.0291117.s006.docx]

**S4 Table. Estimated probability of giving birth to a low-birthweight child, by educational attainment of the mother. Predicted probabilities, adjusted predictions, Margins, 95% CI, Hungary.**

| ***_at edu (1, 2,3)*** | **Delta-method** | | | | | |
| --- | --- | --- | --- | --- | --- | --- |
|  | **Margin** | **Std. Err.** | **z** | **P>\|z\|** | **95% Conf. Interval**  **Lower Upper** | |
| **MODEL 1.** |  |  |  |  |  |  |
| **1. Lower secondary** | 0.053 | 0.004 | 4.980 | 0.000 | 0.046 | 0.060 |
| **2. Upper secondary** | 0.035 | 0.002 | 5.800 | 0.000 | 0.031 | 0.039 |
| **3. Tertiary** | 0.023 | 0.002 | 0.410 | 0.000 | 0.019 | 0.027 |
| **MODEL 2.** |  |  |  |  |  |  |
| **1. Lower secondary** | 0.050 | 0.004 | 3.650 | 0.000 | 0.043 | 0.057 |
| **2. Upper secondary** | 0.035 | 0.002 | 5.750 | 0.000 | 0.030 | 0.039 |
| **3. Tertiary** | 0.024 | 0.002 | 0.160 | 0.000 | 0.019 | 0.029 |
| **MODEL 3.** |  |  |  |  |  |  |
| **1. Lower secondary** | 0.046 | 0.004 | 2.640 | 0.000 | 0.039 | 0.053 |
| **2. Upper secondary** | 0.034 | 0.002 | 5.610 | 0.000 | 0.030 | 0.039 |
| **3. Tertiary** | 0.026 | 0.003 | 0.040 | 0.000 | 0.021 | 0.031 |

*S4 Table Note:* Binary logistic regression analysis, unadjusted and adjusted predicted probabilities/margins. Educational attainment of the pregnant women: 1. lower secondary (ISCED 97: 0, 1, 2, 3C); 2. upper secondary (ISCED 97: 3, 4); 3. tertiary (ISCED 97: 5, 6). Model 1: baby’s sex, whether the baby is a first child, the mother’s age, the height of the mother (meter). Model 2: Model 1 + equivalised household income per capita quantiles. Model 3: Model 2 + maternal drinking and smoking during pregnancy. Only singleton births at more than 33 weeks’ gestation to mothers aged 18 or over were included in all three surveys for these analyses. *Source:* Cohort ’18 – Growing Up in Hungary (2018–2019), own calculation.
